# Tetra-*n*-butyl­ammonium iodido(pyrrolidine-1-carbodithio­ato-κ^2^ *S*,*S*′)(tris-*tert*-butoxy­silanethiol­ato-κ*S*)cadmate(II)

**DOI:** 10.1107/S1600536808014888

**Published:** 2008-05-21

**Authors:** Anna Kropidłowska, Jarosław Chojnacki, Barbara Becker

**Affiliations:** a Department of Inorganic Chemistry, Chemical Faculty, Gdańsk University of Technology, 11/12 G. Narutowicza Str., 80-952 Gdańsk, Poland

## Abstract

In the anion of the title compound, (C_16_H_36_N)[Cd(C_5_H_8_NS_2_)(C_12_H_27_O_3_SSi)I], the Cd atom is four-coordinated by *S*,*S*′-chelating dithio­carbamate, *S*–donating silanethiol­ate and iodide ligands in a distorted tetrahedral environment . Inter­molecular C—H⋯ S and C—H⋯I inter­actions between cations and anions are present. Two C atoms of a *tert*-butyl group are disordered over two positions; the site occupancies are *ca* 0.65 and 0.35.

## Related literature

For related literature, see: Allen (2002 ▶); Battaglia & Corradi (1986 ▶); Clemente *et al.* (1987 ▶); Kropidłowska *et al.* (2006*a* ▶,*b* ▶); Kropidłowska, Chojnacki *et al.* (2008 ▶); Kropidłowska, Rotaru *et al.* (2008 ▶); Wojnowski *et al.* (1992 ▶).
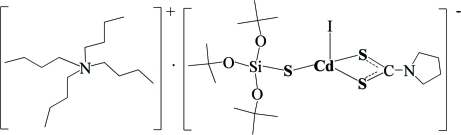


## Experimental

### 

#### Crystal data

(C_16_H_36_N)[Cd(C_5_H_8_NS_2_)(C_12_H_27_O_3_SSi)I]
*M* *_r_* = 907.49
Monoclinic, 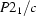

*a* = 14.8881 (9) Å
*b* = 16.3973 (9) Å
*c* = 18.6471 (11) Å
β = 105.519 (5)°
*V* = 4386.3 (5) Å^3^
*Z* = 4
Mo *K*α radiation
μ = 1.40 mm^−1^
*T* = 120 (2) K
0.34 × 0.08 × 0.05 mm

#### Data collection

Oxford Diffraction KM-4-CCD diffractometer
Absorption correction: analytical [*CrysAlis RED* (Oxford Diffraction, 2006 ▶); analytical numeric absorption correction using a multifaceted crystal model based on expressions derived by Clark & Reid (1995 ▶)] *T*_min_ = 0.744, *T*_max_ = 0.943
32652 measured reflections
8628 independent reflections
5398 reflections with *I* > 2σ(*I*)
*R*_int_ = 0.065

#### Refinement

*R*[*F* ^2^ > 2σ(*F* ^2^)] = 0.039
*wR*(*F* ^2^) = 0.088
*S* = 0.86
8628 reflections
423 parameters
H-atom parameters constrained
Δρ_max_ = 1.01 e Å^−3^
Δρ_min_ = −0.44 e Å^−3^

### 

Data collection: *CrysAlis CCD* (Oxford Diffraction, 2006 ▶); cell refinement: *CrysAlis RED* (Oxford Diffraction, 2006 ▶); data reduction: *CrysAlis RED*; program(s) used to solve structure: *SHELXS97* (Sheldrick, 2008 ▶); program(s) used to refine structure: *SHELXL97* (Sheldrick, 2008 ▶); molecular graphics: *ORTEP-3 for Windows* (Farrugia, 1997 ▶) and *Mercury* (Macrae *et al.*, 2006 ▶); software used to prepare material for publication: *WinGX* (Farrugia, 1999 ▶).

## Supplementary Material

Crystal structure: contains datablocks global, I. DOI: 10.1107/S1600536808014888/sg2247sup1.cif

Structure factors: contains datablocks I. DOI: 10.1107/S1600536808014888/sg2247Isup2.hkl

Additional supplementary materials:  crystallographic information; 3D view; checkCIF report

## Figures and Tables

**Table 1:** Hydrogen-bond geometry (Å, °)

*D*—H⋯*A*	*D*—H	H⋯*A*	*D*⋯*A*	*D*—H⋯*A*
C17—H17*A*⋯I1^i^	0.99	3.16	3.830 (4)	126
C30—H30*B*⋯I1^ii^	0.99	3.15	3.960 (4)	139
C31—H31*B*⋯S3	0.99	2.99	3.931 (7)	160

Symmetry codes: (i) 
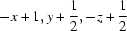
; (ii) 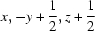.

**Fig. 1.**
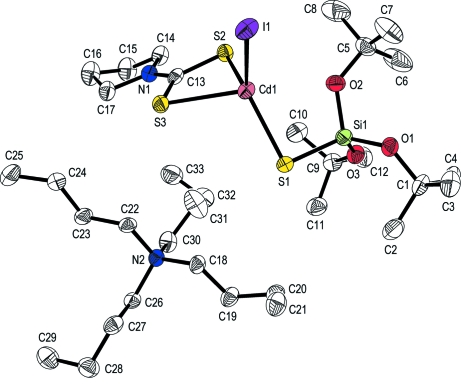
Molecular structure and atom-numbering scheme for [Cd{SSi(OtBu)3}(S2CNC4H9)I](-)[Et4N](+) (I) with displacement ellipsoids drawn at 50% probability level. Atoms C32 and C33 are disordered over two positions - only major set is shown. H atoms have been omitted for clarity.

**Fig. 2.**
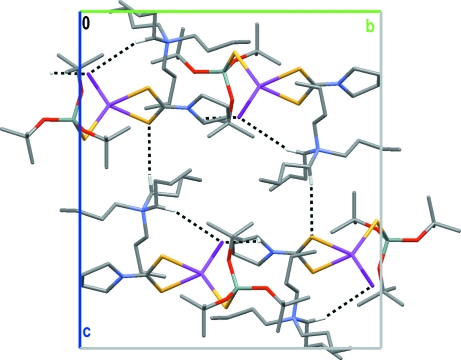
Interactions and short contacts present within the unit cell of I - view along the a axis.

**Fig. 3.**
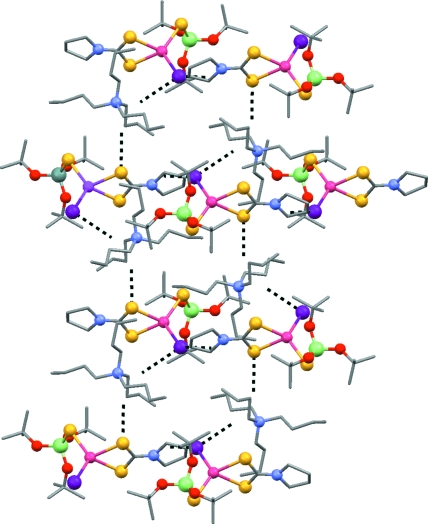
The network of interactions in the crystal lattice of I view along the a axis.

**Fig. 4.**
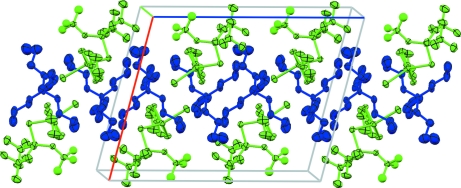
The crystal lattice of I. Color codes: blue-cations; green - anions.

## supplementary crystallographic information

### Comment

The study of metal complexes with S-donor ligands is a well established field. Current interest in such systems stems from the fact that they may serve as potential precursors of  metal sulfides. Our recent investigations are therefore focused on the synthesis of compounds with different S-donors (among them one with S—Si bond) ligating to the same metallic centre (Kropidłowska, Chojnacki, *et al.*, 2008; Kropidłowska, Rotaru, *et al.*, 2008). The synthesis and analysis of products obtained in [Cd{SSi(O*^t^*Bu)_3_}_2_]_2_ (Wojnowski, *et al.*, 1992) | ^(-)^S_2_CN*R*_2_ (dtc) | *R*'_4_NX reaction system have so far proved, that besides the desired complexes with sulfur rich kernel (Kropidłowska, Chojnacki, *et al.*, 2008) some other ionic compounds, *e.g.*, (Et_4_N)[Cd(S_2_NEt_2_)_2_I] (Kropidłowska, *et al.*, 2006*a*) or (Bu_4_N)_2_[CdBr_4_]×2C_7_H_8_ (Kropidłowska, *et al.*, 2006*b*) may also be formed.

In the present paper we describe the product of a reaction between cadmium tri-*tert*-butoxysilanethiolate, ammonium 1-pyrrolidinylcarbodithioate and tetraethylammonium iodide carried out in toluene/propanol-2 mixture. Its crystal structure was determined, demonstrating that a new iodocadmate, [Cd{SSi(O*^t^*Bu)_3_}(S_2_CNC_4_H_9_)I]^(-)^[Et_4_N]^(+)^ (I), with additional silanethiolate and 1-pyrrolidinylcarbodithioate ligands was obtained. The molecular structure of I with atom numbering scheme is shown in Figure 1. The compound represents a novel type of complex with mixed S-donor ligands. Up to now structures (Allen, 2002) of only two similar compounds have been determined, namely for Pt(II) - FISMAV (Clemente *et al.*, 1987) and Bi(III) - DUDWII (Battaglia & Corradi,1986) where dtc ligand and other S-donor (*S*-methyl dtc and thiourea respectively) are accompanied by a halogenide.

In the complex [Cd{SSi(O*^t^*Bu)_3_}(S_2_CNC_4_H_9_)I]^(-) ^anion Cd atoms are four-coordinated in a CdS_3_I core. The arrangement of donor atoms can be primarily regarded as distorted (because of the presence of the chelating agent) tetrahedron with the widest S1—Cd1—S2 (124.78°) angle. Because of the distance (2.782 Å) the additional interaction between Cd1 and O2 is questionable (compare *e.g.* PAGBAA (Wojnowski *et al.*, 1992)). A weak C—H···S interaction between cation and one of the dtc sulfur atoms (S3) can be assumed with d(H···S) 2.99Å (Table 1). What more, also C—H···I short contacts between iodine and some methylene groups from both dtc ligand and cation, with d(H···I) 3.16Å and 3.15Å respectively, may be taken into account (Figure 2). It is worthy to note, that in the unit cell of I cations and anions are arranged in the form of alternating layers (Figure 3).

It is hard to state how iodide ligand enters cadmium coordination sphere. Most probably species possessing both S-donors undergo this reaction. Related investigations are now in progress.

### Experimental

Ammonium 1-pyrrolidinylcarbodithioate (0.029 g, 0.177 mmol) and equimolar amount of tetra-*n*-butylammoniumiodide (0.066 g, 0.178 mmol) were dissolved in 15 cm^3 ^of toluene/propanol-2 (5:1, *v*/*v*) mixture. Next, cadmium tri-*tert*-butoxysilanethiolate (0.11 g, 0.089 mmol, (Wojnowski *et al.*, 1992)) was dissolved in 5 cm^3^of hot toluene/propanol-2 mixture and the solution was added to dithiocarbamate solution. The reagents were stirred and heated under reflux for 4 h under argon and then left for decantation. Clear solution was drained to a Schlenk tube and left at 5°C to crystallize the products. After a week needle-like crystals were collected.

### Refinement

All H atoms were placed in calculated positions and refined as riding on their carrier atoms with respective *U*_iso_(H) values: C—H = 0.98 Å (CH_3_) and *U*_iso_(H) = 1.5 *U*_eq_(C), C—H = 0.99 Å (CH_2_) and *U*_ĩso_(H) = 1.2 *U*_eq_(C). Atoms C32 and C33 within one of *n*-butyl groups (C30—C33) are disordered over two positions with probabilities 0.65/0.35.

### Crystal data

**Table:**

(C_16_H_36_N)[Cd(C_5_H_8_NS_2_)(C_12_H_27_O_3_SSi)I]	*Z* = 4
*M**_r_* = 907.49	*F*_000_ = 1880
Monoclinic, *P*2_1_/*c*	*D*_x_ = 1.374 Mg m^−^^3^
Hall symbol: -P 2ybc	Mo *K*α radiation λ = 0.71073 Å
*a* = 14.8881 (9) Å	θ = 2.5–32.6º
*b* = 16.3973 (9) Å	µ = 1.40 mm^−^^1^
*c* = 18.6471 (11) Å	*T* = 120 (2) K
β = 105.519 (5)º	Needle, colourless
*V* = 4386.3 (5) Å^3^	0.34 × 0.08 × 0.05 mm

### Data collection

**Table:**

Oxford Diffraction KM-4-CCD diffractometer	8628 independent reflections
Monochromator: graphite	5398 reflections with *I* > 2σ(*I*)
Detector resolution: 8.1883 pixels mm^-1^	*R*_int_ = 0.065
*T* = 120(2) K	θ_max_ = 26º
ω scans	θ_min_ = 2.4º
Absorption correction: analytical[CrysAlis RED (Oxford Diffraction, 2006); analytical numeric absorption correction using a multifaceted crystal model based on expressions derived by Clark & Reid (1995)]	*h* = −17→18
*T*_min_ = 0.744, *T*_max_ = 0.943	*k* = −20→19
32652 measured reflections	*l* = −22→23

### Refinement

**Table:**

Refinement on *F*^2^	Secondary atom site location: difference Fourier map
Least-squares matrix: full	Hydrogen site location: inferred from neighbouring sites
*R*[*F*^2^ > 2σ(*F*^2^)] = 0.039	H-atom parameters constrained
*wR*(*F*^2^) = 0.088	*w* = 1/[σ^2^(*F*_o_^2^) + (0.0471*P*)^2^] where *P* = (*F*_o_^2^ + 2*F*_c_^2^)/3
*S* = 0.86	(Δ/σ)_max_ = 0.002
8628 reflections	Δρ_max_ = 1.01 e Å^−^^3^
423 parameters	Δρ_min_ = −0.44 e Å^−^^3^
Primary atom site location: structure-invariant direct methods	Extinction correction: none

### Special details

**Table:**

Geometry. All e.s.d.'s (except the e.s.d. in the dihedral angle between two l.s. planes) are estimated using the full covariance matrix. The cell e.s.d.'s are taken into account individually in the estimation of e.s.d.'s in distances, angles and torsion angles; correlations between e.s.d.'s in cell parameters are only used when they are defined by crystal symmetry. An approximate (isotropic) treatment of cell e.s.d.'s is used for estimating e.s.d.'s involving l.s. planes.
Refinement. Refinement of *F*^2^ against ALL reflections. The weighted *R*-factor *wR* and goodness of fit *S* are based on *F*^2^, conventional *R*-factors *R* are based on *F*, with *F* set to zero for negative *F*^2^. The threshold expression of *F*^2^ > σ(*F*^2^) is used only for calculating *R*-factors(gt) *etc*. and is not relevant to the choice of reflections for refinement. *R*-factors based on *F*^2^ are statistically about twice as large as those based on *F*, and *R*- factors based on ALL data will be even larger.

### Fractional atomic coordinates and isotropic or equivalent isotropic displacement parameters (Å^2^)

**Table:**

	*x*	*y*	*z*	*U*_iso_*/*U*_eq_	Occ. (<1)
Cd1	0.30998 (2)	0.104413 (19)	0.278712 (17)	0.02783 (9)	
N1	0.3153 (2)	0.3675 (2)	0.29473 (19)	0.0284 (9)	
O1	0.14159 (19)	−0.11475 (17)	0.31904 (16)	0.0316 (7)	
O2	0.1439 (2)	0.01702 (18)	0.23839 (16)	0.0334 (7)	
O3	0.0676 (2)	0.01545 (18)	0.35510 (16)	0.0337 (7)	
S2	0.22367 (8)	0.23808 (7)	0.22960 (6)	0.0290 (3)	
S1	0.28545 (8)	0.02356 (7)	0.38426 (6)	0.0299 (3)	
S3	0.41572 (8)	0.23419 (7)	0.33244 (6)	0.0301 (3)	
Si1	0.15373 (8)	−0.01594 (8)	0.32351 (7)	0.0288 (3)	
I1	0.39336 (2)	0.034354 (18)	0.179160 (17)	0.03680 (10)	
C1	0.1167 (3)	−0.1721 (3)	0.3691 (3)	0.0353 (11)	
C2	0.1662 (4)	−0.1504 (3)	0.4499 (3)	0.0458 (13)	
H2A	0.2331	−0.1442	0.4553	0.069*	
H2B	0.1561	−0.194	0.4828	0.069*	
H2C	0.1409	−0.0991	0.4633	0.069*	
C3	0.1497 (4)	−0.2547 (3)	0.3498 (3)	0.0466 (13)	
H3A	0.1195	−0.2673	0.2975	0.07*	
H3B	0.1334	−0.2966	0.3817	0.07*	
H3C	0.2175	−0.2536	0.3577	0.07*	
C4	0.0115 (3)	−0.1730 (3)	0.3562 (3)	0.0487 (14)	
H4A	−0.0101	−0.1181	0.364	0.073*	
H4B	−0.0051	−0.211	0.3911	0.073*	
H4C	−0.0181	−0.1905	0.305	0.073*	
C5	0.0752 (3)	−0.0009 (3)	0.1681 (3)	0.0376 (12)	
C6	−0.0197 (4)	−0.0133 (5)	0.1797 (3)	0.074 (2)	
H6A	−0.0206	−0.0645	0.2067	0.111*	
H6B	−0.0661	−0.016	0.1313	0.111*	
H6C	−0.0344	0.0322	0.2086	0.111*	
C7	0.1083 (4)	−0.0747 (4)	0.1348 (3)	0.0630 (17)	
H7A	0.1736	−0.0672	0.1349	0.094*	
H7B	0.0701	−0.0821	0.0836	0.094*	
H7C	0.1027	−0.123	0.1643	0.094*	
C8	0.0763 (5)	0.0739 (4)	0.1197 (3)	0.076 (2)	
H8A	0.0558	0.1218	0.1424	0.114*	
H8B	0.0341	0.065	0.0701	0.114*	
H8C	0.1397	0.083	0.1153	0.114*	
C9	0.0421 (3)	0.0913 (3)	0.3841 (2)	0.0337 (11)	
C10	0.0733 (4)	0.1643 (3)	0.3458 (3)	0.0437 (13)	
H10A	0.1415	0.1655	0.3579	0.066*	
H10B	0.0508	0.2148	0.3632	0.066*	
H10C	0.0476	0.1596	0.2918	0.066*	
C11	0.0879 (3)	0.0931 (3)	0.4676 (2)	0.0427 (13)	
H11A	0.0657	0.0468	0.4914	0.064*	
H11B	0.0718	0.1442	0.4885	0.064*	
H11C	0.1558	0.0896	0.4766	0.064*	
C12	−0.0637 (3)	0.0905 (3)	0.3688 (3)	0.0479 (14)	
H12A	−0.0922	0.0938	0.315	0.072*	
H12B	−0.0837	0.1373	0.3933	0.072*	
H12C	−0.0834	0.0399	0.3881	0.072*	
C13	0.3184 (3)	0.2873 (3)	0.2869 (2)	0.0261 (10)	
C14	0.2327 (3)	0.4177 (3)	0.2626 (3)	0.0347 (11)	
H14A	0.1761	0.3929	0.2715	0.042*	
H14B	0.2232	0.4247	0.2083	0.042*	
C15	0.2549 (3)	0.4988 (3)	0.3030 (3)	0.0487 (14)	
H15A	0.2252	0.5446	0.2707	0.058*	
H15B	0.2336	0.4994	0.3489	0.058*	
C16	0.3595 (3)	0.5039 (3)	0.3208 (3)	0.0474 (14)	
H16A	0.3845	0.5408	0.3635	0.057*	
H16B	0.3795	0.5239	0.2774	0.057*	
C17	0.3925 (3)	0.4161 (3)	0.3402 (3)	0.0349 (11)	
H17A	0.4512	0.405	0.3266	0.042*	
H17B	0.4021	0.4049	0.3939	0.042*	
N2	0.4647 (2)	0.19938 (19)	0.59242 (18)	0.0249 (8)	
C18	0.4401 (3)	0.1098 (2)	0.5800 (2)	0.0289 (10)	
H18A	0.4986	0.078	0.5892	0.035*	
H18B	0.4057	0.1019	0.5272	0.035*	
C19	0.3819 (3)	0.0751 (3)	0.6284 (3)	0.0341 (11)	
H19A	0.3298	0.1127	0.6281	0.041*	
H19B	0.4208	0.0699	0.6803	0.041*	
C20	0.3430 (3)	−0.0091 (3)	0.5992 (3)	0.0338 (11)	
H20A	0.3051	−0.0302	0.6314	0.041*	
H20B	0.3011	−0.0024	0.5484	0.041*	
C21	0.4168 (3)	−0.0712 (3)	0.5966 (3)	0.0421 (12)	
H21A	0.4612	−0.0755	0.6458	0.063*	
H21B	0.4497	−0.0542	0.5601	0.063*	
H21C	0.3873	−0.1244	0.5822	0.063*	
C22	0.5240 (3)	0.2197 (2)	0.5404 (2)	0.0277 (10)	
H22A	0.5757	0.1798	0.549	0.033*	
H22B	0.4855	0.2121	0.4886	0.033*	
C23	0.5655 (3)	0.3042 (3)	0.5468 (2)	0.0332 (11)	
H23A	0.6056	0.3128	0.5979	0.04*	
H23B	0.5149	0.3452	0.5373	0.04*	
C24	0.6231 (3)	0.3158 (3)	0.4912 (2)	0.0357 (11)	
H24A	0.669	0.2708	0.4973	0.043*	
H24B	0.5814	0.3123	0.4401	0.043*	
C25	0.6744 (4)	0.3961 (3)	0.4999 (3)	0.0544 (15)	
H25A	0.7205	0.3977	0.5485	0.082*	
H25B	0.6299	0.4409	0.4965	0.082*	
H25C	0.706	0.4018	0.4604	0.082*	
C26	0.5175 (3)	0.2158 (3)	0.6728 (2)	0.0271 (10)	
H26A	0.4772	0.1998	0.7049	0.033*	
H26B	0.5284	0.2752	0.6789	0.033*	
C27	0.6106 (3)	0.1726 (3)	0.7009 (2)	0.0327 (11)	
H27A	0.6519	0.1873	0.6693	0.039*	
H27B	0.6008	0.1128	0.6977	0.039*	
C28	0.6570 (4)	0.1966 (3)	0.7818 (3)	0.0413 (12)	
H28A	0.6127	0.1862	0.8119	0.05*	
H28B	0.712	0.1612	0.8012	0.05*	
C29	0.6878 (4)	0.2841 (3)	0.7919 (3)	0.0500 (14)	
H29A	0.6329	0.3197	0.7813	0.075*	
H29B	0.7256	0.297	0.7577	0.075*	
H29C	0.7249	0.2927	0.8433	0.075*	
C30	0.3781 (3)	0.2521 (3)	0.5773 (3)	0.0354 (11)	
H30A	0.3468	0.2422	0.6172	0.042*	
H30B	0.3976	0.31	0.5806	0.042*	
C31	0.3079 (4)	0.2388 (3)	0.5032 (3)	0.0605 (16)	
H31A	0.288	0.181	0.4975	0.073*	
H31B	0.3351	0.2533	0.4619	0.073*	
C32	0.2223 (7)	0.2961 (6)	0.5028 (6)	0.043 (2)	0.646 (11)
H32A	0.1647	0.2731	0.4693	0.052*	0.646 (11)
H32B	0.2137	0.2994	0.5535	0.052*	0.646 (11)
C33	0.2390 (6)	0.3765 (7)	0.4776 (5)	0.057 (3)	0.646 (11)
H33A	0.246	0.3731	0.4269	0.086*	0.646 (11)
H33B	0.2962	0.3989	0.5108	0.086*	0.646 (11)
H33C	0.1863	0.4121	0.4781	0.086*	0.646 (11)
C32A	0.2498 (10)	0.3141 (12)	0.4648 (9)	0.043 (2)	0.354 (11)
H32C	0.2891	0.3632	0.4672	0.052*	0.354 (11)
H32D	0.2163	0.3026	0.4123	0.052*	0.354 (11)
C33A	0.1826 (15)	0.3225 (15)	0.5139 (10)	0.064 (6)	0.354 (11)
H33D	0.1408	0.3688	0.4968	0.097*	0.354 (11)
H33E	0.2184	0.3316	0.5656	0.097*	0.354 (11)
H33F	0.1458	0.2724	0.5109	0.097*	0.354 (11)

### Atomic displacement parameters (Å^2^)

**Table:**

	*U*^11^	*U*^22^	*U*^33^	*U*^12^	*U*^13^	*U*^23^
Cd1	0.03032 (19)	0.02514 (18)	0.02964 (18)	−0.00049 (15)	0.01080 (14)	0.00300 (14)
N1	0.027 (2)	0.027 (2)	0.031 (2)	0.0019 (17)	0.0088 (17)	0.0000 (16)
O1	0.0297 (17)	0.0344 (18)	0.0331 (18)	−0.0072 (14)	0.0127 (14)	−0.0026 (14)
O2	0.0301 (17)	0.044 (2)	0.0260 (17)	−0.0020 (15)	0.0079 (14)	0.0019 (14)
O3	0.0327 (18)	0.0352 (19)	0.0368 (18)	−0.0021 (14)	0.0157 (14)	−0.0017 (14)
S2	0.0277 (6)	0.0257 (6)	0.0322 (6)	0.0001 (5)	0.0053 (5)	0.0009 (5)
S1	0.0329 (6)	0.0317 (7)	0.0246 (6)	−0.0063 (5)	0.0067 (5)	0.0031 (5)
S3	0.0291 (6)	0.0280 (6)	0.0304 (6)	−0.0029 (5)	0.0032 (5)	0.0043 (5)
Si1	0.0270 (7)	0.0339 (8)	0.0271 (7)	−0.0037 (6)	0.0098 (5)	0.0004 (5)
I1	0.0454 (2)	0.02957 (17)	0.04242 (19)	0.00608 (15)	0.02381 (15)	0.00383 (14)
C1	0.041 (3)	0.025 (3)	0.044 (3)	−0.005 (2)	0.018 (2)	0.004 (2)
C2	0.056 (3)	0.040 (3)	0.045 (3)	−0.005 (3)	0.019 (3)	0.009 (2)
C3	0.045 (3)	0.040 (3)	0.057 (3)	−0.009 (3)	0.018 (3)	−0.004 (3)
C4	0.039 (3)	0.041 (3)	0.075 (4)	−0.009 (2)	0.029 (3)	0.001 (3)
C5	0.030 (3)	0.049 (3)	0.030 (3)	−0.001 (2)	0.002 (2)	−0.002 (2)
C6	0.034 (3)	0.142 (6)	0.045 (3)	−0.011 (4)	0.007 (3)	−0.019 (4)
C7	0.062 (4)	0.072 (4)	0.048 (4)	0.007 (3)	0.002 (3)	−0.024 (3)
C8	0.084 (5)	0.087 (5)	0.041 (4)	−0.001 (4)	−0.013 (3)	0.015 (3)
C9	0.033 (3)	0.039 (3)	0.035 (3)	0.003 (2)	0.018 (2)	−0.004 (2)
C10	0.056 (3)	0.034 (3)	0.046 (3)	0.006 (3)	0.023 (3)	0.000 (2)
C11	0.048 (3)	0.047 (3)	0.037 (3)	−0.001 (3)	0.019 (2)	0.001 (2)
C12	0.047 (3)	0.052 (3)	0.047 (3)	−0.005 (3)	0.017 (3)	−0.010 (3)
C13	0.030 (2)	0.024 (2)	0.030 (2)	−0.0016 (19)	0.017 (2)	0.0008 (19)
C14	0.034 (3)	0.028 (3)	0.045 (3)	0.001 (2)	0.015 (2)	−0.001 (2)
C15	0.046 (3)	0.036 (3)	0.066 (4)	0.001 (2)	0.018 (3)	−0.007 (3)
C16	0.047 (3)	0.035 (3)	0.065 (4)	−0.002 (2)	0.024 (3)	−0.012 (3)
C17	0.033 (3)	0.034 (3)	0.041 (3)	−0.005 (2)	0.015 (2)	−0.007 (2)
N2	0.030 (2)	0.0176 (19)	0.028 (2)	−0.0002 (15)	0.0099 (16)	−0.0040 (15)
C18	0.036 (3)	0.019 (2)	0.032 (3)	−0.004 (2)	0.011 (2)	−0.0033 (19)
C19	0.041 (3)	0.030 (3)	0.037 (3)	−0.003 (2)	0.021 (2)	−0.002 (2)
C20	0.037 (3)	0.033 (3)	0.032 (3)	−0.003 (2)	0.012 (2)	0.002 (2)
C21	0.047 (3)	0.032 (3)	0.050 (3)	−0.003 (2)	0.017 (3)	−0.004 (2)
C22	0.038 (3)	0.027 (2)	0.020 (2)	−0.002 (2)	0.011 (2)	−0.0016 (18)
C23	0.045 (3)	0.025 (3)	0.029 (3)	−0.004 (2)	0.009 (2)	0.0035 (19)
C24	0.038 (3)	0.038 (3)	0.030 (3)	−0.006 (2)	0.007 (2)	0.004 (2)
C25	0.068 (4)	0.050 (3)	0.048 (3)	−0.022 (3)	0.020 (3)	0.007 (3)
C26	0.037 (3)	0.027 (2)	0.022 (2)	−0.004 (2)	0.015 (2)	−0.0053 (19)
C27	0.044 (3)	0.021 (2)	0.035 (3)	0.001 (2)	0.015 (2)	0.002 (2)
C28	0.046 (3)	0.041 (3)	0.035 (3)	−0.001 (2)	0.006 (2)	−0.002 (2)
C29	0.056 (4)	0.050 (3)	0.040 (3)	−0.006 (3)	0.008 (3)	−0.012 (2)
C30	0.039 (3)	0.025 (3)	0.045 (3)	0.001 (2)	0.015 (2)	−0.007 (2)
C31	0.047 (3)	0.045 (3)	0.078 (4)	0.014 (3)	−0.003 (3)	−0.017 (3)
C32	0.035 (5)	0.057 (6)	0.042 (6)	0.006 (4)	0.018 (4)	−0.008 (4)
C33	0.048 (6)	0.075 (9)	0.045 (5)	−0.002 (5)	0.006 (4)	0.005 (5)
C32A	0.035 (5)	0.057 (6)	0.042 (6)	0.006 (4)	0.018 (4)	−0.008 (4)
C33A	0.061 (14)	0.098 (17)	0.041 (10)	0.006 (12)	0.025 (10)	−0.014 (10)

### Geometric parameters (Å, °)

**Table:**

Cd1—S1	2.4798 (11)	C17—H17A	0.99
Cd1—S2	2.5815 (11)	C17—H17B	0.99
Cd1—S3	2.6770 (11)	N2—C22	1.513 (5)
Cd1—I1	2.7459 (4)	N2—C30	1.516 (5)
N1—C13	1.326 (5)	N2—C18	1.517 (5)
N1—C14	1.467 (5)	N2—C26	1.518 (5)
N1—C17	1.468 (5)	C18—C19	1.518 (6)
O1—C1	1.441 (5)	C18—H18A	0.99
O1—Si1	1.630 (3)	C18—H18B	0.99
O2—C5	1.460 (5)	C19—C20	1.539 (6)
O2—Si1	1.646 (3)	C19—H19A	0.99
O3—C9	1.446 (5)	C19—H19B	0.99
O3—Si1	1.631 (3)	C20—C21	1.509 (6)
S2—C13	1.726 (4)	C20—H20A	0.99
S1—Si1	2.0880 (16)	C20—H20B	0.99
S3—C13	1.709 (4)	C21—H21A	0.98
C1—C3	1.517 (6)	C21—H21B	0.98
C1—C4	1.519 (6)	C21—H21C	0.98
C1—C2	1.532 (6)	C22—C23	1.509 (6)
C2—H2A	0.98	C22—H22A	0.99
C2—H2B	0.98	C22—H22B	0.99
C2—H2C	0.98	C23—C24	1.524 (6)
C3—H3A	0.98	C23—H23A	0.99
C3—H3B	0.98	C23—H23B	0.99
C3—H3C	0.98	C24—C25	1.509 (6)
C4—H4A	0.98	C24—H24A	0.99
C4—H4B	0.98	C24—H24B	0.99
C4—H4C	0.98	C25—H25A	0.98
C5—C6	1.500 (7)	C25—H25B	0.98
C5—C7	1.503 (7)	C25—H25C	0.98
C5—C8	1.526 (7)	C26—C27	1.519 (6)
C6—H6A	0.98	C26—H26A	0.99
C6—H6B	0.98	C26—H26B	0.99
C6—H6C	0.98	C27—C28	1.532 (6)
C7—H7A	0.98	C27—H27A	0.99
C7—H7B	0.98	C27—H27B	0.99
C7—H7C	0.98	C28—C29	1.502 (7)
C8—H8A	0.98	C28—H28A	0.99
C8—H8B	0.98	C28—H28B	0.99
C8—H8C	0.98	C29—H29A	0.98
C9—C12	1.524 (6)	C29—H29B	0.98
C9—C11	1.525 (6)	C29—H29C	0.98
C9—C10	1.529 (6)	C30—C31	1.510 (7)
C10—H10A	0.98	C30—H30A	0.99
C10—H10B	0.98	C30—H30B	0.99
C10—H10C	0.98	C31—C32A	1.566 (18)
C11—H11A	0.98	C31—C32	1.581 (10)
C11—H11B	0.98	C31—H31A	0.99
C11—H11C	0.98	C31—H31B	0.99
C12—H12A	0.98	C32—C33	1.444 (14)
C12—H12B	0.98	C32—H32A	0.99
C12—H12C	0.98	C32—H32B	0.99
C14—C15	1.521 (6)	C33—H33A	0.98
C14—H14A	0.99	C33—H33B	0.98
C14—H14B	0.99	C33—H33C	0.98
C15—C16	1.505 (7)	C32A—C33A	1.53 (2)
C15—H15A	0.99	C32A—H32C	0.99
C15—H15B	0.99	C32A—H32D	0.99
C16—C17	1.533 (6)	C33A—H33D	0.98
C16—H16A	0.99	C33A—H33E	0.98
C16—H16B	0.99	C33A—H33F	0.98
			
S1—Cd1—S2	124.78 (4)	C16—C17—H17A	111.2
S1—Cd1—S3	108.95 (4)	N1—C17—H17B	111.2
S2—Cd1—S3	69.20 (3)	C16—C17—H17B	111.2
S1—Cd1—I1	120.14 (3)	H17A—C17—H17B	109.1
S2—Cd1—I1	112.80 (3)	C22—N2—C30	111.6 (3)
S3—Cd1—I1	104.99 (3)	C22—N2—C18	105.9 (3)
C13—N1—C14	124.2 (4)	C30—N2—C18	111.4 (3)
C13—N1—C17	123.8 (4)	C22—N2—C26	110.9 (3)
C14—N1—C17	111.9 (3)	C30—N2—C26	105.8 (3)
C1—O1—Si1	131.3 (3)	C18—N2—C26	111.2 (3)
C5—O2—Si1	131.3 (3)	N2—C18—C19	115.2 (3)
C9—O3—Si1	135.8 (3)	N2—C18—H18A	108.5
C13—S2—Cd1	86.03 (14)	C19—C18—H18A	108.5
Si1—S1—Cd1	92.96 (5)	N2—C18—H18B	108.5
C13—S3—Cd1	83.34 (14)	C19—C18—H18B	108.5
O1—Si1—O3	104.18 (15)	H18A—C18—H18B	107.5
O1—Si1—O2	107.28 (16)	C18—C19—C20	110.3 (4)
O3—Si1—O2	111.67 (16)	C18—C19—H19A	109.6
O1—Si1—S1	114.29 (12)	C20—C19—H19A	109.6
O3—Si1—S1	114.92 (12)	C18—C19—H19B	109.6
O2—Si1—S1	104.45 (12)	C20—C19—H19B	109.6
O1—C1—C3	105.9 (4)	H19A—C19—H19B	108.1
O1—C1—C4	109.7 (4)	C21—C20—C19	114.1 (4)
C3—C1—C4	109.8 (4)	C21—C20—H20A	108.7
O1—C1—C2	110.3 (4)	C19—C20—H20A	108.7
C3—C1—C2	109.8 (4)	C21—C20—H20B	108.7
C4—C1—C2	111.2 (4)	C19—C20—H20B	108.7
C1—C2—H2A	109.5	H20A—C20—H20B	107.6
C1—C2—H2B	109.5	C20—C21—H21A	109.5
H2A—C2—H2B	109.5	C20—C21—H21B	109.5
C1—C2—H2C	109.5	H21A—C21—H21B	109.5
H2A—C2—H2C	109.5	C20—C21—H21C	109.5
H2B—C2—H2C	109.5	H21A—C21—H21C	109.5
C1—C3—H3A	109.5	H21B—C21—H21C	109.5
C1—C3—H3B	109.5	C23—C22—N2	116.6 (3)
H3A—C3—H3B	109.5	C23—C22—H22A	108.1
C1—C3—H3C	109.5	N2—C22—H22A	108.1
H3A—C3—H3C	109.5	C23—C22—H22B	108.1
H3B—C3—H3C	109.5	N2—C22—H22B	108.1
C1—C4—H4A	109.5	H22A—C22—H22B	107.3
C1—C4—H4B	109.5	C22—C23—C24	110.7 (4)
H4A—C4—H4B	109.5	C22—C23—H23A	109.5
C1—C4—H4C	109.5	C24—C23—H23A	109.5
H4A—C4—H4C	109.5	C22—C23—H23B	109.5
H4B—C4—H4C	109.5	C24—C23—H23B	109.5
O2—C5—C6	111.1 (4)	H23A—C23—H23B	108.1
O2—C5—C7	107.7 (4)	C25—C24—C23	113.3 (4)
C6—C5—C7	112.0 (5)	C25—C24—H24A	108.9
O2—C5—C8	104.7 (4)	C23—C24—H24A	108.9
C6—C5—C8	110.8 (5)	C25—C24—H24B	108.9
C7—C5—C8	110.2 (5)	C23—C24—H24B	108.9
C5—C6—H6A	109.5	H24A—C24—H24B	107.7
C5—C6—H6B	109.5	C24—C25—H25A	109.5
H6A—C6—H6B	109.5	C24—C25—H25B	109.5
C5—C6—H6C	109.5	H25A—C25—H25B	109.5
H6A—C6—H6C	109.5	C24—C25—H25C	109.5
H6B—C6—H6C	109.5	H25A—C25—H25C	109.5
C5—C7—H7A	109.5	H25B—C25—H25C	109.5
C5—C7—H7B	109.5	N2—C26—C27	116.2 (3)
H7A—C7—H7B	109.5	N2—C26—H26A	108.2
C5—C7—H7C	109.5	C27—C26—H26A	108.2
H7A—C7—H7C	109.5	N2—C26—H26B	108.2
H7B—C7—H7C	109.5	C27—C26—H26B	108.2
C5—C8—H8A	109.5	H26A—C26—H26B	107.4
C5—C8—H8B	109.5	C26—C27—C28	110.5 (4)
H8A—C8—H8B	109.5	C26—C27—H27A	109.6
C5—C8—H8C	109.5	C28—C27—H27A	109.6
H8A—C8—H8C	109.5	C26—C27—H27B	109.6
H8B—C8—H8C	109.5	C28—C27—H27B	109.6
O3—C9—C12	106.5 (4)	H27A—C27—H27B	108.1
O3—C9—C11	108.0 (4)	C29—C28—C27	114.3 (4)
C12—C9—C11	110.4 (4)	C29—C28—H28A	108.7
O3—C9—C10	110.9 (3)	C27—C28—H28A	108.7
C12—C9—C10	110.3 (4)	C29—C28—H28B	108.7
C11—C9—C10	110.6 (4)	C27—C28—H28B	108.7
C9—C10—H10A	109.5	H28A—C28—H28B	107.6
C9—C10—H10B	109.5	C28—C29—H29A	109.5
H10A—C10—H10B	109.5	C28—C29—H29B	109.5
C9—C10—H10C	109.5	H29A—C29—H29B	109.5
H10A—C10—H10C	109.5	C28—C29—H29C	109.5
H10B—C10—H10C	109.5	H29A—C29—H29C	109.5
C9—C11—H11A	109.5	H29B—C29—H29C	109.5
C9—C11—H11B	109.5	C31—C30—N2	115.7 (4)
H11A—C11—H11B	109.5	C31—C30—H30A	108.4
C9—C11—H11C	109.5	N2—C30—H30A	108.4
H11A—C11—H11C	109.5	C31—C30—H30B	108.4
H11B—C11—H11C	109.5	N2—C30—H30B	108.4
C9—C12—H12A	109.5	H30A—C30—H30B	107.4
C9—C12—H12B	109.5	C30—C31—C32A	117.8 (8)
H12A—C12—H12B	109.5	C30—C31—C32	106.1 (5)
C9—C12—H12C	109.5	C30—C31—H31A	110.5
H12A—C12—H12C	109.5	C32A—C31—H31A	126.3
H12B—C12—H12C	109.5	C32—C31—H31A	110.5
N1—C13—S3	120.1 (3)	C30—C31—H31B	110.5
N1—C13—S2	119.0 (3)	C32A—C31—H31B	75.3
S3—C13—S2	120.9 (2)	C32—C31—H31B	110.5
N1—C14—C15	103.9 (4)	H31A—C31—H31B	108.7
N1—C14—H14A	111	C33—C32—C31	109.6 (9)
C15—C14—H14A	111	C33—C32—H32A	109.8
N1—C14—H14B	111	C31—C32—H32A	109.8
C15—C14—H14B	111	C33—C32—H32B	109.8
H14A—C14—H14B	109	C31—C32—H32B	109.8
C16—C15—C14	103.5 (4)	H32A—C32—H32B	108.2
C16—C15—H15A	111.1	C33A—C32A—C31	99.6 (14)
C14—C15—H15A	111.1	C33A—C32A—H32C	111.9
C16—C15—H15B	111.1	C31—C32A—H32C	111.9
C14—C15—H15B	111.1	C33A—C32A—H32D	111.9
H15A—C15—H15B	109	C31—C32A—H32D	111.9
C15—C16—C17	104.4 (4)	H32C—C32A—H32D	109.6
C15—C16—H16A	110.9	C32A—C33A—H33D	109.5
C17—C16—H16A	110.9	C32A—C33A—H33E	109.5
C15—C16—H16B	110.9	H33D—C33A—H33E	109.5
C17—C16—H16B	110.9	C32A—C33A—H33F	109.5
H16A—C16—H16B	108.9	H33D—C33A—H33F	109.5
N1—C17—C16	102.8 (4)	H33E—C33A—H33F	109.5
N1—C17—H17A	111.2		
			
S1—Cd1—S2—C13	94.76 (14)	Cd1—S3—C13—S2	−7.3 (2)
S3—Cd1—S2—C13	−4.40 (14)	Cd1—S2—C13—N1	−173.1 (3)
I1—Cd1—S2—C13	−102.47 (14)	Cd1—S2—C13—S3	7.5 (2)
S2—Cd1—S1—Si1	71.18 (6)	C13—N1—C14—C15	165.6 (4)
S3—Cd1—S1—Si1	148.54 (5)	C17—N1—C14—C15	−11.2 (5)
I1—Cd1—S1—Si1	−90.42 (5)	N1—C14—C15—C16	29.8 (5)
S1—Cd1—S3—C13	−116.52 (14)	C14—C15—C16—C17	−37.3 (5)
S2—Cd1—S3—C13	4.46 (14)	C13—N1—C17—C16	171.6 (4)
I1—Cd1—S3—C13	113.57 (14)	C14—N1—C17—C16	−11.6 (5)
C1—O1—Si1—O3	−40.7 (4)	C15—C16—C17—N1	30.1 (5)
C1—O1—Si1—O2	−159.2 (3)	C22—N2—C18—C19	178.6 (4)
C1—O1—Si1—S1	85.6 (4)	C30—N2—C18—C19	−59.8 (5)
C9—O3—Si1—O1	168.0 (4)	C26—N2—C18—C19	58.0 (5)
C9—O3—Si1—O2	−76.5 (4)	N2—C18—C19—C20	167.2 (4)
C9—O3—Si1—S1	42.2 (4)	C18—C19—C20—C21	58.8 (5)
C5—O2—Si1—O1	47.7 (4)	C30—N2—C22—C23	63.9 (5)
C5—O2—Si1—O3	−65.9 (4)	C18—N2—C22—C23	−174.6 (4)
C5—O2—Si1—S1	169.4 (3)	C26—N2—C22—C23	−53.8 (5)
Cd1—S1—Si1—O1	123.88 (12)	N2—C22—C23—C24	−180.0 (4)
Cd1—S1—Si1—O3	−115.73 (13)	C22—C23—C24—C25	−174.2 (4)
Cd1—S1—Si1—O2	6.96 (13)	C22—N2—C26—C27	−56.0 (4)
Si1—O1—C1—C3	−160.4 (3)	C30—N2—C26—C27	−177.3 (4)
Si1—O1—C1—C4	81.2 (5)	C18—N2—C26—C27	61.6 (4)
Si1—O1—C1—C2	−41.6 (5)	N2—C26—C27—C28	178.3 (4)
Si1—O2—C5—C6	34.7 (6)	C26—C27—C28—C29	−67.5 (5)
Si1—O2—C5—C7	−88.3 (5)	C22—N2—C30—C31	66.4 (5)
Si1—O2—C5—C8	154.3 (4)	C18—N2—C30—C31	−51.9 (5)
Si1—O3—C9—C12	153.6 (3)	C26—N2—C30—C31	−172.9 (4)
Si1—O3—C9—C11	−87.8 (5)	N2—C30—C31—C32A	−147.3 (7)
Si1—O3—C9—C10	33.6 (6)	N2—C30—C31—C32	176.6 (5)
C14—N1—C13—S3	−175.3 (3)	C30—C31—C32—C33	85.9 (8)
C17—N1—C13—S3	1.2 (5)	C32A—C31—C32—C33	−29.8 (12)
C14—N1—C13—S2	5.4 (5)	C30—C31—C32A—C33A	−74.3 (14)
C17—N1—C13—S2	−178.2 (3)	C32—C31—C32A—C33A	4.1 (11)
Cd1—S3—C13—N1	173.3 (3)		

### Hydrogen-bond geometry (Å, °)

**Table:**

*D*—H···*A*	*D*—H	H···*A*	*D*···*A*	*D*—H···*A*
C17—H17A···I1^i^	0.99	3.16	3.830 (4)	126
C30—H30B···I1^ii^	0.99	3.15	3.960 (4)	139
C31—H31B···S3	0.99	2.99	3.931 (7)	160

Symmetry codes: (i) −*x*+1, *y*+1/2, −*z*+1/2; (ii) *x*, −*y*+1/2, *z*+1/2.
